# Cross-cultural adaptation of the Digital Health Literacy Instrument (DHLI) for use on Brazilian adolescents

**DOI:** 10.1590/0103-6440202305346

**Published:** 2023-12-22

**Authors:** Mariane Carolina Faria Barbosa, Ana Luiza Peres Baldiotti, Náyra Santos Braga, Camila Takáo Lopes, Saul Martins Paiva, Ana Flávia Granville-Garcia, Fernanda de Morais Ferreira

**Affiliations:** 1 Departament of Pediatric Dentistry, Federal University of Minas Gerais(UFMG), Belo Horizonte, (MG), Brazil.; 2 Paulista School of Nursing, Federal University of São Paulo(UNIFESP), São Paulo, (SP) Brazil.; 3 Postgraduate Program in Dentistry, State University of Paraíba(UEPB), Campina Grande, (PB), Brazil.

**Keywords:** Adolescent, Health Literacy, Cross-Cultural Comparison, Internet, eHealth Strategies

## Abstract

The present study aimed to perform the cross-cultural adaptation of the Digital Health Literacy Instrument (DHLI) for native Brazilian Portuguese-speaking adolescents (DHLI-BrA). Cross-cultural adaptation consisted of the following steps: translation, assessment, and adjustments by the expert committee to ensure cultural equivalence; back-translation, and synthesis of back-translations. Cognitive testing was then performed in a pretest with adolescents using cognitive interviews with probing questions on the item's understanding interpretation and response options. Cronbach’s alpha coefficient and McDonald’s omega were used to estimate the instrument’s reliability. Forty-two Brazilian adolescents participated in the study (mean age: 16.0 ± 2.0 years; range: 13 to 19 years). Items that were difficult to understand were adapted to the context of Brazilian adolescents. Cronbach’s alpha coefficient and McDonald’s omega for the 21 items of the DHLI-BrA were, respectively, 0.79 and 0.80. Cronbach’s alpha coefficient for the subscales of the self-report instrument was 0.53-0.79 (range), demonstrating good reliability in the total instrument and moderate reliability in the subscales. This study provides the cross-cultural adapted version of the Digital Health Literacy Instrument (DHLI), which is an instrument for measuring digital Health literacy, for use in Brazilian adolescents (DHLI-BrA).

## Resumo

O estudo teve o objetivo de adaptar transculturalmente o Digital Health Literacy Instrument (DHLI) para adolescentes nativos do idioma português do Brasil (DHLI-BrA). O estudo de adaptação transcultural consistiu nas seguintes etapas: tradução, avaliação e adequação de equivalência cultural da tradução por comitê de especialistas; retrotradução e síntese das retrotraduções. Foi realizada a testagem cognitiva em pré-teste com adolescentes, utilizando-se entrevistas cognitivas com perguntas de sondagem sobre a compreensão e interpretação dos itens e opções de resposta. O alfa de *Cronbach* e ômega de McDonald’s foram utilizados para estimar a confiabilidade do instrumento. Participaram do pré-teste 42 adolescentes brasileiros com média de idade de 16,0 ± 2,0 (variação de 13-19) anos. Os itens com dificuldade de compreensão foram adaptados ao contexto dos adolescentes brasileiros. O coeficiente alfa de *Cronbach* e o ômega de *McDonald’s*, para os 21 itens do DHLI-BrA foi respectivamente, 0,79 e 0,80. O coeficiente alfa de Cronbach para as subescalas do instrumento de autorrelato foi de 0,53-0,79 (variação), demonstrando boa confiabilidade no instrumento total e confiabilidade moderada nas subescalas. Este estudo fornece a versão adaptada transculturalmente do *Digital Health Literacy Instrument* (DHLI), um instrumento de mensuração do letramento digital em saúde, para utilização em adolescentes brasileiros (DHLI-BrA).

## Introduction

Digital media connect people to vital resources, such as education, income, and health ^1^,^2^. An estimated 4.9 billion people throughout the world have access to the Internet ^3^. In Brazil, 82.7% of homes have an internet connection, and 90.2% of Brazilian adolescents are connected ^4^. The digitalization of healthcare services has changed in recent years and digital devices, such as smartphones, laptops, and wearable devices, have become essential to the health field ^5^,^6^. Most adolescents in Brazil access the internet through smartphones ^4^. This means of access promotes changes in the use of online health resources, as smartphone devices enable instant access from any location ^7^.

Adolescents, digital natives, spend up to one-quarter of their daily time accessing different media ^8^,^9^ and have a high level of competency in using the technologies ^8^. Adolescence is characterized by neurobiological, cognitive, and social development, as well as increased self-awareness and an interest in one's health body care, and well-being ^8^,^10^,^11^,^12^. To better understand and manage their health, teenagers often look for information and the internet is an attractive resource, due to the easy access to a wide range of health topics and the possible search for sensitive information anonymously ^8^,^13^. A study found that 84% of US adolescents reported having used the internet at least once in their lifetime to access health information ^13^. Nevertheless, adolescents do not always find it easy to use the health information they find on the web, as they can present a low health literacy ^8^,^14^.

However, given the large quantity of information, which is often imprecise or of low quality, individuals need to have skills that go beyond the instrumental domain of technological devices ^1^,^15^,^16^. Therefore, adolescents need to have skills for researching, selecting, assessing, interpreting, and applying online health information for their benefit or to solve a health problem. Such skills are denominated digital health literacy (DHL) ^5^,^17^. Based on these premises, instruments have been developed for the assessment of research skills and the use of health information in the digital environment self-reported by healthcare users ^17^,^18^.

With technological advances and the interactivity of digital media, one should take a broad spectrum of skills into account in the current concept of eHealth, such as the ability to write and post messages related to health on the web, self-manage one’s own health and chronic conditions with the use of applications and use telehealth services (Health 2.0) ^5^,^16^,^19^. Thus, the Digital Health Literacy Instrument (DHLI) was developed, a self-report instrument that measures the complete spectrum of eHealth skills (Health 1.0 and Health 2.0), including actual competencies ^5^. This instrument has been translated and culturally adapted for use in Brazil and demonstrated adequate construct validity for adults with chronic diseases ^20^.

The use of instruments that enable considering the patient’s perspective - patient-reported outcome measures (PROMs) - has the potential to assist in the evaluation of the patient’s evolution, improve communication between health care providers and patients, and assist in improving health practices and services. However, to ensure the psychometric validity of PROMs it is necessary to consider the target population for which the instrument was validated and perform necessary adaptations for use in other age groups and cultural contexts ^21^. Considering these premises, the high degree of digital connectedness among Brazilian adolescents, the particularities of this age group, and the absence of an instrument for measuring DHL in this group, the present study aimed to perform the cross-cultural adaptation of the DHLI for native Brazilian Portuguese-speaking adolescents.

## Methods

### Description of instrument

The Digital Health Literacy Instrument (DHLI) was originally developed in English and Dutch and is a self-report scale with 21 items that address the broad spectrum of the eHealth concept, including the use of health information on the internet (Health 1.0) and recent applications with interactive technologies (Health 2.0) (5). The DHLI addresses seven skills, each with three items: 1. Operational skills (using a computer and surfing the internet); 2. Navigation skills (finding your way around the web); 3. Information search (using appropriate search strategies); 4. Evaluate the reliability of the information found; 5. Determine the relevance of online information; 6. Add self-generated content; and 7. Protect and respect privacy on the Internet ^5^.

Each item is answered individually and is scored on a four-point scale (1 to 4), with response options ranging from "very easy" to "very difficult" and from "never" to "almost always". To calculate the total score, at least 18 items need to be answered and the average of the item scores is calculated (very easy/never = 4; reasonably easy/sometimes = 3; reasonably difficult/often = 2; very difficult/almost always = 1). Higher scores denote a higher level of DHL. Specific scores can also be calculated per skill by the average of the scores of the three items referring to each skill on the instrument ^5^.

The DHLI has seven additional items that address practical performance for each of the seven skills that compose the instrument. These seven items can be applied in digital or print form and have five response options - one correct option (score = 1), three incorrect options (score = 0), and "I don’t know" (score = 0). To calculate the total DHL score based on performance, at least six of the seven items must be answered ^5^.

### Study design and ethical aspects

A methodological cross-cultural adaptation study was conducted with Brazilian adolescents in the period from February to June 2022. Individuals between 13 and 19 years of age enrolled at three public schools in the city of Belo Horizonte, Brazil, were invited to participate.

This study received approval from the Human Research Ethics Committee of the Federal University of Minas Gerais (process number: 51689627.1.0000.5149; protocol: 5.073.552). All legal guardians and adolescents of 18 or 19 years of age signed a consent form and those under 18 years of age agreed to participate by signing an assent form.

### Cross-cultural adaptation

This study was conducted after authorization from the DHLI original authors (5). A universal approach was adopted for translation and cross-cultural adaptation ^22^,^23^. The manuscript was written using the COSMIN checklist referring to the cross-cultural adaptation process ^24^. The following steps were performed to achieve this study's proposed objectives: translation, assessment, and adjustments by the expert committee (to ensure cultural equivalence); back-translations and synthesis of back-translations; cognitive testing (pretest) with adolescents involving cognitive interviews with probing questions.

### Translation

The original instrument was translated into Brazilian Portuguese by two independent Brazilian translators proficient in English. Then, a third Brazilian translator formulated a synthesis of the two translations.

## Expert committee

The Brazilian version of the DHLI was submitted to an expert committee of native Brazilian Portuguese-speaking specialists with a background in the health field, expertise in health literacy, experience in cross-cultural adaptation, and validation of research instruments for use on adolescents. This step was conducted to determine 1) conceptual equivalence to ensure the original instrument theoretic concept maintenance of the theoretic concept proposed by the original instrument, 2) semantic equivalence to ensure the meaning of the words in terms of vocabulary and grammar and 3) item equivalence - analysis of items adequacy of the original instrument for the assessment of DHL in the target culture.

Two meetings were held between the main researchers and members of the committee to assess equivalence with the original instrument as well as determine the clarity and adequacy of the items and language for the adolescent age group. The members of the committee could suggest changes to the words/expressions and images of the instrument if deemed necessary. This process led to the initial version of the DHLI adapted for Brazilian adolescents (DHLI-AV1).

### Back-translation

The DHLI-AV1 was back-translated into English by two independent native English-speaking translators with linguistic mastery of English and Portuguese and no awareness of the original instrument or the objectives of the study.

A committee of three Brazilian specialists with a background in the health field and fluent in English evaluated the two back-translated versions and made comparisons to the original instrument to obtain a synthesis back-translated version in English. The synthesis version was sent for the original instrument author’s assessment, who considered the proposed changes to be appropriate. Therefore, it was not necessary to perform any additional changes to the DHLI-AV1 in this phase.

### Pretest

The first pretest of the DHLI-AV1 was performed with a non-probabilistic convenience sample of 28 adolescents 13 to 19 years of age. A sample was selected of individuals with the representativeness of the instrument's target population and distributed similarly to the Brazilian population in terms of age, sex, and internet access characteristics ^4^. The purpose of this step was to determine the instrument's applicability and understanding of the items by the participants. The adolescents were encouraged to point out difficulties and suggest changes for words/expressions that were difficult to understand as well as propose the rewriting of items. The participants were also encouraged to evaluate the layout questionnaire organization.

Adolescents who met the following requirements were included: aged 13 to 19 years, native speakers of Brazilian Portuguese, and having regular access to the internet. The exclusion criteria were vision, hearing, or cognitive problems (self-reported or informed by the school) that disabled participation in the study.

The instrument was administered individually in a standardized manner in print form in a reserved room at the schools. For sample characterization, the participants also answered a questionnaire addressing sociodemographic characteristics and questions about access and search for information on the Internet. A cognitive interview was held immediately after the questionnaires involving the use of probing questions conducted by one of the researchers (M.C.F.B.). The first pretest was concluded when saturation of all items was reached.

A committee of three specialists with a background in the health field performed a qualitative assessment of all questions, suggestions, and comments made by the adolescents during the first pretest. All adjustments that did not alter the conceptual meaning of the original item were performed, giving rise to the second version of the adapted instrument (DHLI-AV2), which was presented to 14 different adolescent volunteers. The method of the second pretest was the same as that used for the first pretest.

### Statistical analysis

Data descriptive analysis was performed using *SPSS Statistics* 21.0 (IBM Corp., Armonk, N.Y., USA). The reliability of the instrument’s final version was determined based on internal consistency, which was estimated using Cronbach’s alpha coefficient. Values > 0.61 were considered substantial and adequate ^25^.

## Results

### Cross-cultural adaptation of self-report scale

Box 1 1displays the results of the cross-cultural adaptation of the 21 self-report items of the DHLI for use on Brazilian adolescents. The first column is the items of the original version in English ^5^, followed by the initial and final versions for Brazilian adolescents.

**Box 1:**
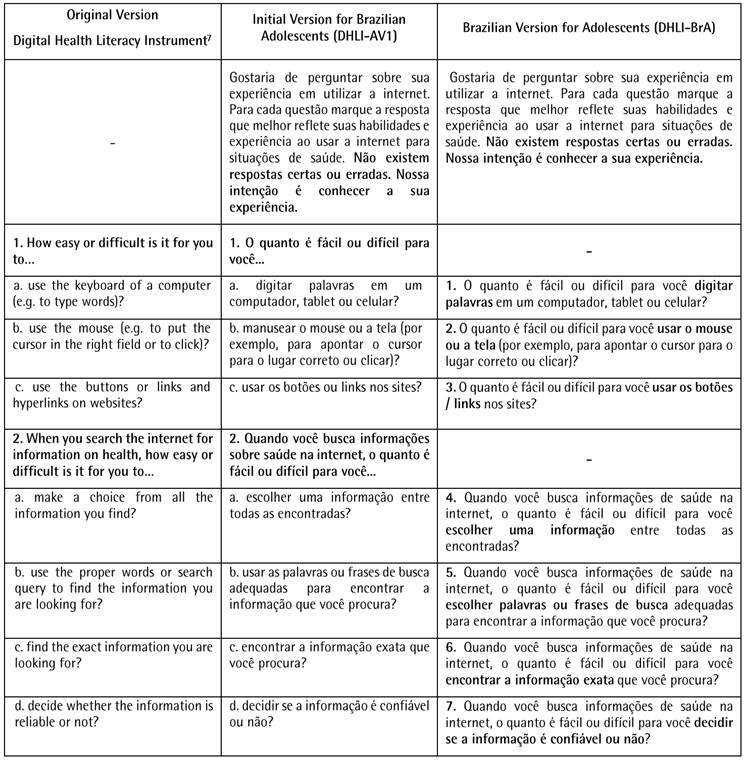
Original Version Digital Health Literacy Instrument, Initial Version, and Brazilian Version for Adolescents of the Digital Health Literacy Instrument (DHLI-BrA).

**Box 1:**
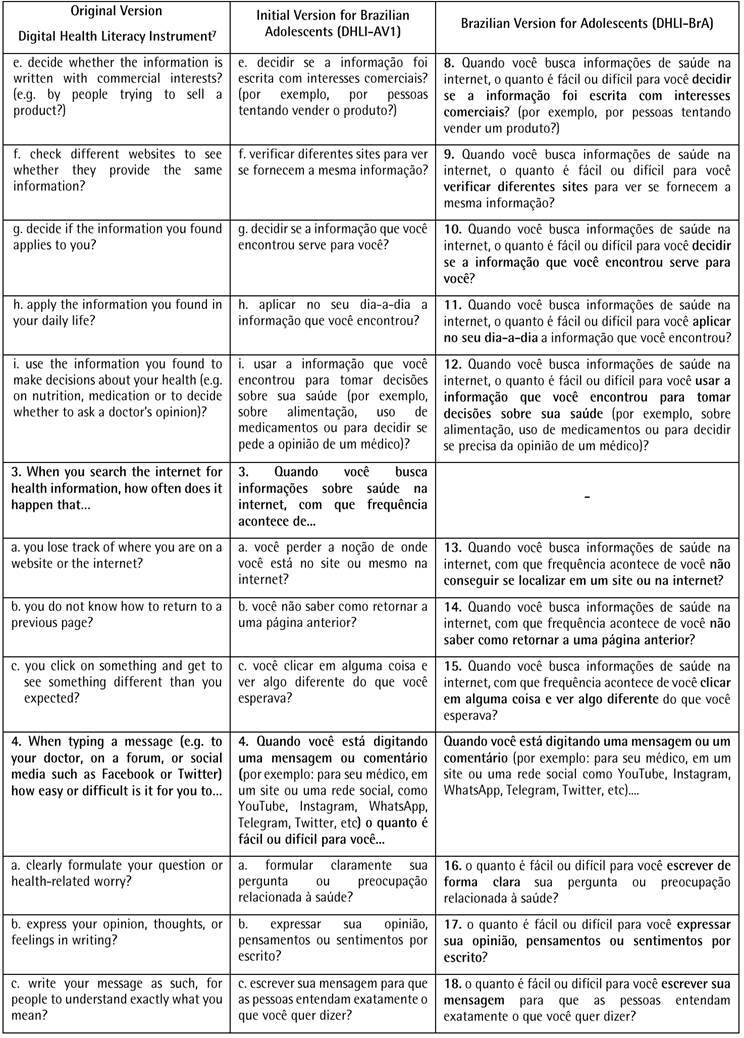
Continuation

**Box 1:**
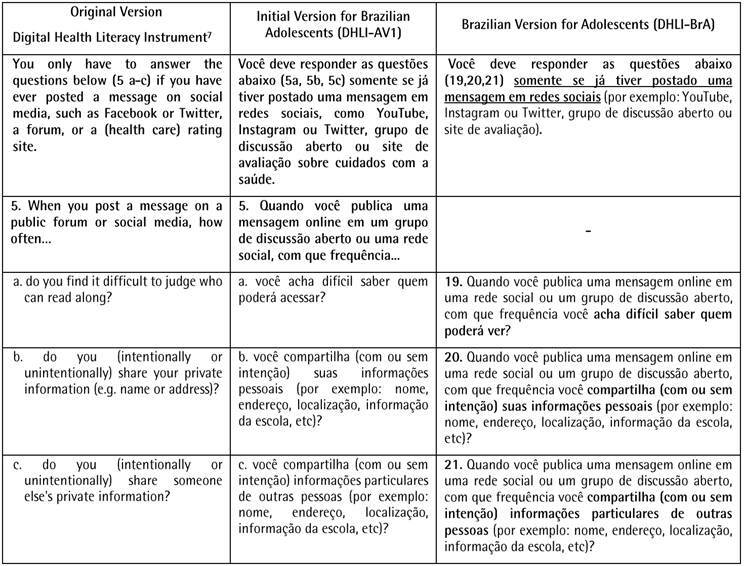
Continuation

Based on the specialist’s suggestions, some consensual changes were made to the version of the DHLI translated into Brazilian Portuguese. The changes began with the inclusion of a statement to facilitate the adolescents’ understanding. At this stage, the need to adapt the instrument, which is aimed at the use of computers, to the Brazilian context was verified, in which smartphones are the main means of access to the internet, especially in this age group. In this regard, two items were adjusted - 1a “use the keyboard of a computer (i.e., type words)” was changed to “enter words on a computer, tablet or cell phone” and 1b “use the mouse...” was changed to “use the mouse or screen...”.

The specialists suggested changing some terms considered formal or difficult to understand, such as “pages on the internet” to “sites”, “nutrition” to “eating”; “private information” to “personal information” and “forum” to “group discussions”. Moreover, the decision was made to remove the term "hyperlink" and update examples of social media to those currently most used by adolescents in Brazil (*Instagram* and *TikTok*).

During the back-translation step, the level of agreement between the two back-translated versions of the self-report items was 72.5%. The expert committee considered both back-translations to have small or moderate divergences. Back-translation 1 was declared more accurate in terms of correspondence to the original instrument. Divergences were resolved by consensus of the committee of the three specialists, who then developed the synthesis back-translated version.

During the first and second pretests, the Brazilian version of the instrument (DHLI-BrA) proved to be easy to administer, and the average time required to answer the self-report items was 4.97 ± 2.63 minutes. In the first pretest, difficulties were observed with the original layout of the instrument, in which the initial part of each item is located in the statement. To solve this problem, text was added to the statements for each item, and the overall numbering of the instrument was changed, which ended up being 1 to 21. Considering comparisons with other studies and the compilation of multiple databanks, the order of the items on the original instrument was maintained.

The difficulty was detected in understanding some of the terms. Thus, “handle the mouse...” was changed to “use the mouse...”; “use words...” was changed to “choose words...”; “you lose the notion of where you are on the site or even on the internet?” was changed to “you are unable to locate where you are on a site or the internet?”; “clearly formulate...” was changed to “write clearly..."; "...who might access?” was changed to “...who might see?”. Another suggestion presented by the adolescents was the use of bold type for important words in each of the items. All adaptations proved effective, as no further changes were deemed necessary after the second pretest. Thus, the Brazilian version of the Digital Health Literacy Instrument for Adolescents (DHLI-BrA) was obtained.

Table 1 displays the participant’s characteristics in the instrument pretest. A total of 42 adolescents were included (22 males and 20 females, with a mean age of 16.0 ± 2.0 years and mean household income of R$ 2215.08 ± 1301.89). All participants had access to the internet, mainly via a smartphone, and used social media (100%).

**Table 1:** Characteristics of the adolescents who participated in the instrument pre-test and of the DHLI-BrA score (mean and standard deviation) (n=42). Brazil. 2022.

Variable	PT1 n (%)	PT2 n (%)
Sex		
Female	14 (50.0)	6 (42.9)
Male	14 (50.0)	8 (57.1)
Age		
≤ 15	12 (42.9)	6 (42.9)
> 16 ┤19	16 (57.2)	8 (57.1)
Adolescent’s education level		
< 8 years of study	24 (85.7)	11 (78.6)
≥ 8 years of study	4 (14.3)	3 (21.4)
Skin color (self-declared)		
Black	7 (25.0)	6 (42.9)
White	10 (35.7)	3 (21.4)
Brown	10 (35.7)	5 (35.7)
Indigenous	1 (3.6)	0 (0.0)
Parents/guardians education level		
< 8 years of study	11 (47.8)	5 (64.3)
≥ 8 years of study	12 (52.2)	8 (61.5)
Health of the adolescent (self-reported)		
Bad/Fair	3 (10.7)	5 (35.7)
Good/Very good	25 (89.3)	9 (64.3)
Oral health of the adolescent (self-reported)		
Bad/Fair	9 (32.1)	4 (28.6)
Good/Very good	19 (67.9)	10 (71.4)
Ability to use the internet (self-reported)		
Bad/Fair	5 (17.9)	0 (0.0)
Good/Very good	23 (82.1)	14 (100.0)
Looking for health information on the internet		
Yes	24 (85.7)	13 (92.9)
No	4 (14.3)	1 (7.1)
Uses health-related smartphone app		
Yes	7 (25.0)	2 (14.3)
No	21 (75.0)	12 (85.7)
Do you have a Computer/Notebook/Tablet?		
Yes	14 (50.0)	4 (28.6)
No	14 (50.0)	10 (71.4)
Internet access frequency		
Every day	24 (85.7)	12 (85.7)
Almost everyday	4 (14.3)	2 (14.3)
Did you follow guidelines/health tips from bloggers? digital influencers or people you follow on the social network?		
Yes	13 (46.4)	8 (57.1)
No	15 (53.6)	6 (42.9)
Do you look for information about a doctor/dentist on social networks before consulting?		
Yes	6 (21.4)	2 (14.3)
No	22 (78.6)	12 (85.7)
Performed self-medication based on information available on the internet		
Yes	21 (75.0)	3 (21.4)
No	7 (25.0)	11 (78.6)

PT1- First Pretest / PT2 - Second Pretest

Table 2 displays the mean overall score of the self-report items on the scale as well as the mean of each skill.

**Table 2:** Total and subscale scores of self-report items on the DHLI-BrA (n=42).

Subscales	Mean (DP)
Total Scores	3.07 (0.39)
Operational skills	3.68 (0.43)
Navigation skills	3.09 (0.61)
Information searching	2.86 (0.62)
Evaluating the reliability	2.82 (0.73)
Determining the relevance	3.08 (0.68)
Adding self-generated	3.38 (0.62)
Protecting and respecting privacy	3.09 (0.68)*

* N=41

In the analysis of internal consistency, Cronbach’s alpha coefficient and McDonald’s omega, for the 21 self-report items of the DHLI-BrA were, respectively, 0.79 and 0.80, which can be considered indicative of substantial reliability ^25^. Cronbach alphas of the subscales were moderate or substantial, ranging from 0.53 to 0.79. This general coefficient is similar to that of the original instrument (α = 0.87) ^5^. Moreover, no significant change in the alpha coefficient was found when a question was removed.

### Cross-cultural adaptation of seven performance-based items

The expert committee performed changes to the seven items addressing performance-based skills. The screenshot images of the computer were replaced and the statement items were changed to address topics related to the 13 to 19-year-old age group. Three versions of these items were presented: one similar to the original instrument with computer screenshots and two versions with smartphone screenshots according to the main operational systems used in Brazil (Android and iOS). This adaptation was based on the context of the Brazilian population, in which few homes have a computer and the main means of access to the internet is via smartphones ^4^, especially in the target age group. It was necessary to develop two adaptations for distinct operational systems due to differences in functioning and layouts. Standardization was performed of the topics and sites used in the versions for desktop computers, smartphones with the *Android* operational system, and smartphones with the *iOS* operational system.

On Items 1 and 2, the screenshots of the Search for Health Establishment site were replaced with the Brazilian Health Ministry site referring to the influenza vaccination campaign. Item 4, which addressed breastfeeding, was changed to a dermatological topic related to acne. No changes altered the original objective of the instrument. A few changes were made to other items regarded the use of different terms and expressions (e.g., “my husband” to “my father” and “your neighbor” to “your friend”).

During the first and second pretests, the seven performance-based items served for the adolescents and the topics were part of their socio-cultural context. The average time required to complete these items was 9.33 ± 2.53 minutes. Participants who did not have a computer or laptop preferred the version of the instrument directed at the operational system of their smartphone. Only two terms generated difficulties and were adjusted: “minimize this page” to “diminish this page” and “diagnosis” to “identify your condition”. For Item 4, it was necessary to include an explanation for the term dermatology (“a medical specialty that treats skin conditions”). All changes to the seven performance-based items proved to be effective, as the second pretest revealed no need for further modifications.

It was possible to calculate performance-based digital health literacy by the sum of the scores of the seven performance items (range: 0 to 7 points). The participants obtained a mean score of 3.46 ± 1.57 points (range: 1 to 6.5) for performance-based DHL. Table 3 describes the number and percentage of correct answers for each item.

The final version of the DHLI-BrA, with 21 self-report items and three versions for the seven performance-based items (computer, *Android*, and *iOS*) is available in the supplementary material.

**Table 3:** Number and percentage of participants who correctly answered performance-based items (n=42 ).

Subscales	Right answer (%)
Total Scores	27 (64.3)
Operational skills	31 (73.8)
Navigation skills	18 (42.9)
Information searching	19 (45.2)
Evaluating the reliability	17 (40.5)
Determining the relevance	25 (61.0)
Adding self-generated	17 (40.5)

## Discussion

This is the first study to perform the adaptation of a tool for measuring digital health literacy for use in adolescents in Brazil. The purpose of the Digital Health Literacy Instrument ^5^, which was developed and validated for adults in the Netherlands, is to measure the broad spectrum of abilities involved in the concept of eHealth. This instrument has previously been culturally adapted for American adolescents ^16^ and university students of other countries ^26^-^29^, demonstrating low cost and easy application, which is in agreement with the present adaptation.

A universal approach for health instruments was followed for cross-cultural adaptation in the present study ^22^,^23^. The assessment of the expert committee enabled correcting imprecisions and adjusting items to the Brazilian Portuguese language as well as the cross-cultural context and target age group. Due to the rapid evolution of the digital world, some terms were out-of-date and may no longer reflect the current scenario. Thus, some items were adapted, such as the inclusion of the term "screen" and the adaptation of the performance-based items due to the predominance of the use of smartphones ^4^.

The process of cross-cultural adaptation of DHLI-BrA for Brazilian adolescents got an instrument with simple clear language and colloquial expressions, which proved to be pertinent to its purpose of measuring Digital Health Literacy (30). Furthermore, the procedures ensured conceptual, semantic, item, operational, and cross-cultural equivalence ^22^. The preliminary values of Cronbach’s alpha coefficient (0,79) and McDonald’s omega (0,80) indicate that the instrument tends to have good properties, which will be better evaluated later with the psychometric analyses. Thus, after additional testing, the DHLI-BrA will enable the generalization and comparability of the results to those of other socio-cultural and linguistic contexts ^22^,^23^.

The DHLI-BrA for use on adolescents in Brazil can provide information on vulnerable subgroups that face challenges regarding health care in the digital medium. On the individual level, this instrument can provide information to guide and train patients who need assistance in the use of web-based health tools to achieve better outcomes regarding their health. Moreover, the seven performance-based items of the DHLI-BrA can be used independently to assess performance-based DHL by the main form of access to the internet used by adolescents. This instrument can be useful in future studies and used for the diagnosis of adolescents with low DHL and vulnerability in eHealth.

The availability of this research tool can contribute to the expansion of studies on this subject and, consequently, provide a basis for the planning of eHealth promotion strategies in adolescence. Furthermore, it can assist health organizations in the development and adaptation of technologies directed at groups with low DHL, thereby reducing disparities related to the eHealth of the population.

A limitation of the present study was the use of a convenience sample from only three public schools in the same city to perform cross-cultural adaptation for adolescents. Psychometric studies involving the determination of reliability based on test-retest stability, dimensional structure, internal structure, and other variables (criterion validity) should be conducted and are currently underway by our research group to complement the cross-cultural adaptation of the DHLI-BrA.

## Conclusion

This study provides the cross-cultural adapted version of the Digital Health Literacy Instrument (DHLI), which is an instrument for measuring digital Health literacy, for use in Brazilian adolescents (DHLI-BrA).
